# Ion irradiation induced phase transformation in gold nanocrystalline films

**DOI:** 10.1038/s41598-020-74779-2

**Published:** 2020-10-20

**Authors:** Pranav K. Suri, James E. Nathaniel, Nan Li, Jon K. Baldwin, Yongqiang Wang, Khalid Hattar, Mitra L. Taheri

**Affiliations:** 1 Department of Materials Science and Engineering, Drexel University, Philadelphia, PA USA; 2 Department of Materials Science and Engineering, Johns Hopkins University, Baltimore, MD USA; 3 MPA Division, Center for Integrated Nanotechnologies, Los Alamos National Laboratory, Los Alamos, NM USA; 4 MST-8, Los Alamos National Laboratory, Los Alamos, NM USA; 5 Sandia National Laboratories, Albuquerque, NM USA; 6 Present Address: Micron Technology, Inc., Boise, ID USA

**Keywords:** Condensed-matter physics, Materials for energy and catalysis, Nanoscale materials, Structural materials

## Abstract

Gold is a noble metal typically stable as a solid in a face-centered cubic (FCC) structure under ambient conditions; however, under particular circumstances aberrant allotropes have been synthesized. In this work, we document the phase transformation of 25 nm thick nanocrystalline (NC) free-standing gold thin-film via in situ ion irradiation studied using atomic-resolution transmission electron microscopy (TEM). Utilizing precession electron diffraction (PED) techniques, crystallographic orientation and the radiation-induced relative strains were measured and furthermore used to determine that a combination of surface and radiation-induced strains lead to an FCC to hexagonal close packed (HCP) crystallographic phase transformation upon a 10 dpa radiation dose of Au^4+^ ions. Contrary to previous studies, HCP phase in nanostructures of gold was stabilized and did not transform back to FCC due to a combination of size effects and defects imparted by damage cascades.

## Introduction

Over the past two decades, nanomaterials have attracted much attention due to their exceptional properties compared to their coarse-grained and bulk counterparts. Mechanical, electrical, optical, and physical properties such as allotropic phase stability and melting temperature have been shown to be significantly influenced by the increase in interface density associated with decreasing grain sizes into the nanoscale^1^. Interfacial forces such as surface strain readily come into picture in nanomaterials, which can lead to the atomic reorganization of surfaces to minimize their energy^2^. Metals such as chromium, molybdenum, and tungsten, which are normally body-centered cubic (BCC) in structure, have been observed retaining a face-centered cubic (FCC) structure in particles less than 30 nm in diameter^3–5^. Nanoparticles, nanowires, and nanosheets of noble metals have also been found to have different crystal structures due to size effects and furthermore can exhibit material properties differing from their archetypal structural analogs such as enhanced magnetization or conductivity^6–10^. FCC and HCP (hexagonal close packed) ruthenium nanoparticles have been reported to show different size dependences of the activity in CO oxidation reactions due to differences in electronic state and surfaces in the crystal structures^11^.

Similar to ruthenium, HCP gold nanostructures have been found to exhibit catalytic properties differing from FCC gold nanostructures, however stabilizing gold in the HCP phase has been a challenge^7^. High pressure research on elemental gold found that the noble metal maintained its typical FCC structure at pressures up to 180 GPa, however at pressures exceeding 230 GPa at room temperature HCP was detected via X-ray diffraction, yet upon decompression and/or heating transformation back to FCC ensued^8^. However, on several occasions it has been observed that defects can stabilize metastable phases: In reports of as deposited, pulse laser deposited nanocrystalline (NC) nickel (Ni) thin-films containing both FCC and HCP structures undergoing self-ion bombardment it was found that the size and number of HCP grains increased upon irradiation of the specimen^12,13^. HCP phase in NC Ni thin-films was found to be highly dependent on the crystal orientation, the ability to form certain defects in that particular orientation, and on interfacial effects in thin-films of NC metal.

In this work, the transformation and stabilization of an FCC-HCP phase change is studied in free-standing NC gold thin-films irradiated with Au^4+^ ions at 200 °C. High-resolution transmission electron microscopy (HR-TEM) and precession electron diffraction (PED) was used to show that there is an energetic threshold that is overcome by interfacial and radiation-induced strain which can cause a phase transition in gold nanostructures within a thickness greater than shown in previous studies^14^. Deformation mechanisms are presented, and furthermore, the introduction of crystal defects by way of ion bombardment is proposed as a novel route to stabilize metastable phases having exceptional properties uncharacteristic of the typical equilibrium microstructure.

## Results and discussion

**Figure 1:**
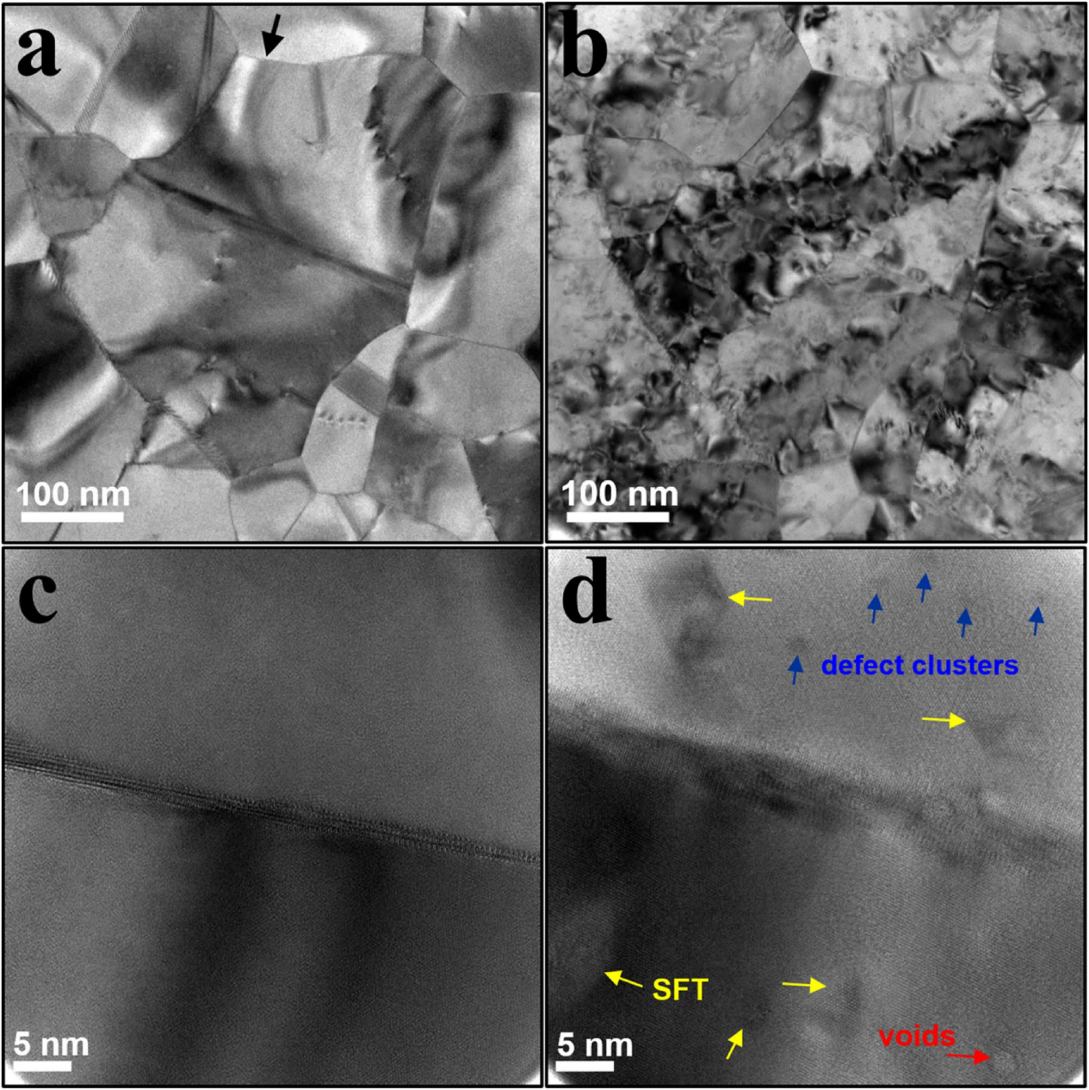
Microstructural evolution of the free-standing gold thin-film before and after ion irradiation. Bright-field CTEM image of the ROI in the gold film (**a**) before and (**b**) after ion irradiation of 10 dpa. Black arrow in (**a**) depicts the higher-magnification bright-field CTEM image of a grain boundary (**c**) before and (**d**) after ion irradiation of 10 dpa. Post-irradiation defects of stacking fault tetrahedra (SFT), defect clusters and voids are respectively labelled with yellow, blue and red arrows in (**d**).

The microstructural evolution of the free-standing gold thin-film before and after ion irradiation is depicted in Fig. 1. Figure 1a shows a bright-field conventional TEM (CTEM) image of the post-anneal microstructure of a region of interest (ROI) containing a ~ 350 nm FCC grain at room temperature (RT); the same ROI is shown in Fig. 1b post-irradiation to 10 dpa at 200 °C. Annealing produced a pristine nanocrystalline structure with minimal visible defect clusters. Significant structural modifications including substantial diffraction contrast and the presence of radiation induced defects is observed as a result of the ion bombardment. Knock-on damage produced by cascades of defects created by the high energy particles impinging on the specimen result in the displacement and the imperfect reorganization of lattice atoms. Mobile defects, if they do not recombine, can agglomerate and form radiation-induced defect clusters. Figure 1c,d are high-resolution images taken next to the same grain boundary before and after irradiation, respectively; the arrows in Fig. 1d highlight defects such as stacking fault tetrahedron (SFT), dislocations, and voids that were not present before irradiation. The formation of SFTs is expected upon irradiation in many FCC metals due to their relatively low stacking fault energies (SFE). Molecular dynamics simulations have shown that SFTs form by direct collapse of a region depleted of atoms during the cascade event into a tetrahedron shaped by four intersecting {111} vacancy stacking faults^15^. SFTs can also form by the agglomeration and rearrangement of vacancies during high-temperature annealing. For most FCC metals, SFTs are more stable than voids as cluster size increases^16^, and in low SFE metals such as Au and Cu the total energy of SFTs is less than Frank and perfect loops as well^17^. The combination of the films $\left\langle {111} \right\rangle$ texture and gold’s SFE of 30–50 mJ/m^2^ over a temperature range of − 273 to 617 °C^18^ facilitate the radiation induced formation of stacking faults^19^.

**Figure 2:**
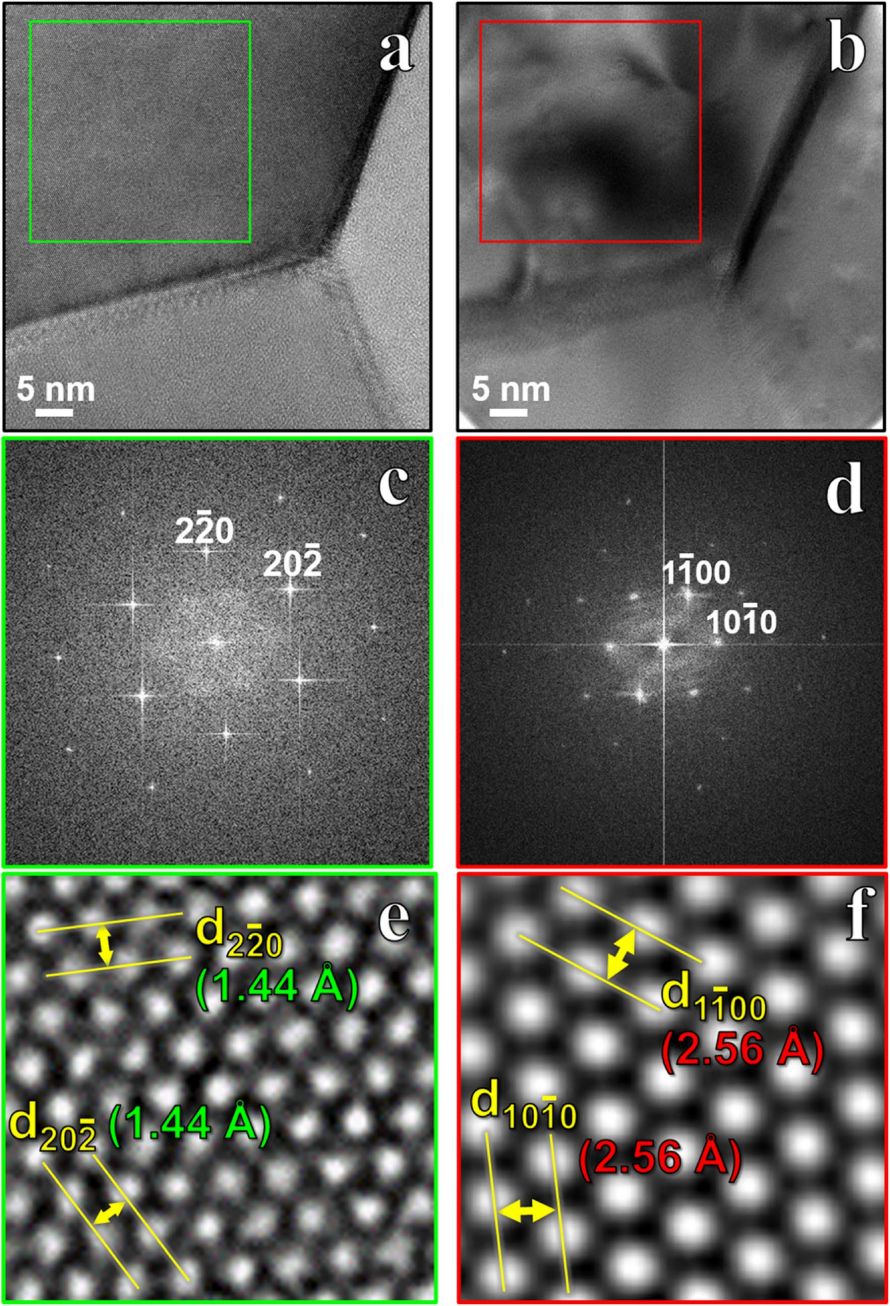
High-resolution microscopy before and after ion irradiation. Bright-field CTEM images from the same region of gold film (**a**) before and (**b**) after irradiation of 10 dpa. Green box in (**a**) denotes the region for the calculation of (**c**,**e**), and the red box in (**b**) denotes the region for the calculation of (**d**,**f**). (**c**) Fourier-transform calculated from the green box in panel (**a**). (**d**) Fourier-transform calculated from the red box in (**b**). (**e**) Bright-field atomic-resolution CTEM image from the green box in (**a**). The *d*-spacings of particular $({{2\overline{2}0) }}$ and $({{20\overline{2}) }}$ planes in FCC gold are labeled. (**f**) Bright-field atomic-resolution CTEM image from the red box in (**a**). The *d*-spacings of particular $({{1\overline{1}00) }}$ and $({{10\overline{1}0) }}$ planes in HCP gold are labeled.

Upon post-irradiation characterization it was found that the crystal orientation and lattice parameters of some grains no longer matched that of an FCC lattice but fit the previously reported parameters of HCP gold^10^. To resolve the variance, 32 grains were analyzed in the ROI with PED and HR-TEM, 6 of which retained the FCC structure and the remaining 26 had transformed to HCP; there was no correlation found between grain size and crystal phase. The structural transformation effects of irradiation are accentuated via atomic-resolution microscopy and image processing in Fig. 2. Figure 2a,b show pre- and post-irradiation high-resolution micrographs and corresponding Fourier-filtered images (Fig. 2c,d) of the grain matrix of a $\left\langle {111} \right\rangle$ grain. When indexing the diffractograms from the heavily deformed grains, previous symmetry of 60° angles from [111] FCC diffraction spots had shifted 30° to angles associated with HCP along [0001]. Figure 2e,f illustrate the differences in *d*-spacings in the grain before and after irradiation. Pre-irradiation there was a 1.44 Å spacing between (2$\overline{2}$0) and (20$\overline{2}$) planes; post-irradiation the atomic structure had shifted such that the spacing between adjacent planes was 2.56 Å, equivalent to the spacings between (10$\overline{1}0$) and (1$\overline{1}00$) planes in an HCP lattice^10^. Note that in the atomic-resolution TEM images, there is no FCC-HCP interface observed. However, HR-TEM and PED patterns from individual grains occasionally showed the presence of FCC phase reflections inside HCP grains and vice-a-versa, denoting an incomplete phase transformation along the direction of electron beam (Fig. S1 in Supplementary Information).

In abnormal grain growth studies on pulse laser deposited NC Ni, researchers conjectured that grains identified having a HCP structure were heavily faulted FCC grains^12,20^; the authors expounded upon how the $\left\langle {111} \right\rangle$ texture is conducive to the formation of stacking faults and hence facilitates the formation of HCP grains with $\left\langle {0001} \right\rangle$ texture^12^. In the present study, grain orientation seems to influence, which grains exhibit HCP structures; grains that had transformed to HCP exhibited a pre-irradiation out-of-plane orientation parallel or near parallel to the (111) orientation with respect to the sample surface. This orientation relationship of {111}_FCC_||{0001}_HCP_ has been documented in previous phase transformation^12,21^ and ion bombardment^22^ studies of NC Ni. The basal planes of the HCP structure are parallel to the (111) matrix planes and the close-packed directions in the two structures are parallel; {111}_FCC_||{0001}_HCP_ is the most common FCC-HCP orientation relationship reported in literature^23^ as to be expected being that these two structures have nearly identical nearest-neighbor distances.

In a number of thin-film deposition and grain growth studies where the transformation of FCC to HCP was observed, it was proposed that highly disordered structures subjected substantial internal stress within the matrix playing an important role in the phase transformation^21,24,25^. Numerous ion bombardment studies have also reported stress-induced phase transformations not based on chemical effects^26–28^; of particular relevance to the work conveyed here, Rajasekhara et al.^22^, Bykov et al*.*^29^, Teodorescu et al*.*^30^, and Johnson et al*.*^31^ articulate accounts of FCC to HCP irradiation-induced transformations in Ni. Hartley^32^ reports that stresses due to the formation of point and extended defects following Ar^+^ and N^+^ implantation can reach extremely high levels (~ 10^4^–10^5^ kg/cm^2^), magnitudes comparable to the yield stress measured in steels.

**Figure 3:**
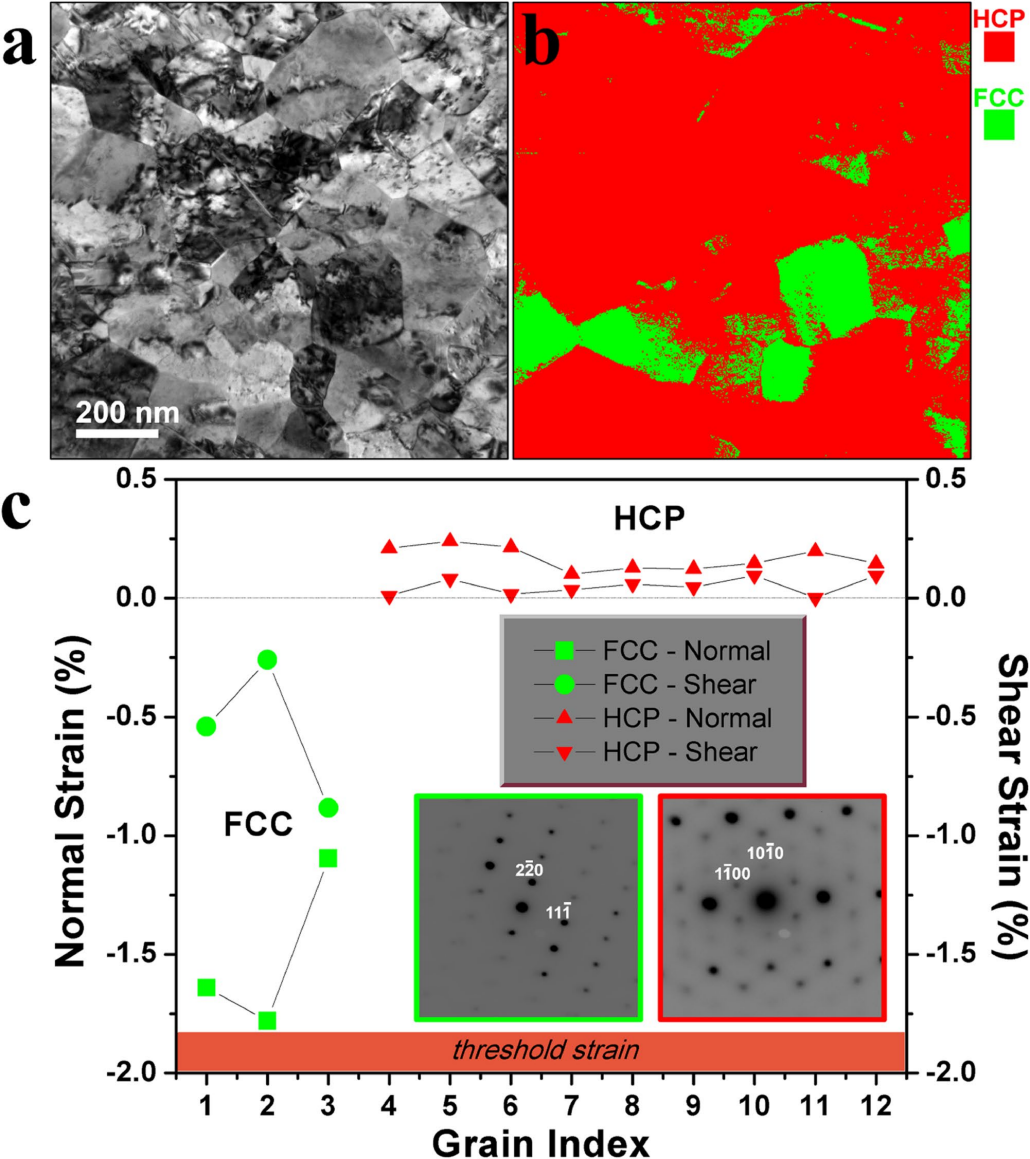
Post irradiation crystal structure analysis. Phase mapping and strain calculation via PED. (**a**) Bright-field CTEM image after ion irradiation of 10 dpa from the ROI. (**b**) PED phase map from the region shown in (**a**) with HCP and FCC phases being marked in red and green, respectively. (**c**) Average normal strain (Max [ε_xx_, ε_yy_]) and shear strain (ε_xy_) plotted for the HCP and FCC phases. Inset shows the representative PED patterns from FCC and HCP phases used to calculate strain and are outlined with green and red colors, respectively. Due to the resolution of PED technique for strain calculation, standard error bars are smaller than the symbol size used.

Here the possibility of irradiation produced deformation-induced, stress-driven phase transformation is explored by estimating a threshold strain for the phase transformation of NC gold as both HCP and FCC grains are present after ion irradiation. In this work, PED is foremost used to confirm the HCP phase formation and complimented by strain measurements in HCP and FCC grains (Fig. 3). PED was used to measure the differences in relative strain in FCC grains in the sample at the pristine state and FCC and HCP grains after irradiation. Strain measurement via PED works best when crystal is oriented close to a zone axis^33^; the $\left\langle {111} \right\rangle$ out-of-plane texture of FCC grains and $\left\langle {0001} \right\rangle$ out-of-plane texture of HCP grains facilitated the experiment. When measuring strain in the specimen in the irradiated state*,* the average strain was measured in regions inside of grains which were relatively less deformed identified by the absence of strong diffraction contrast. Figure 3 shows a micrograph (Fig. 3a) and corresponding phase map (Fig. 3b) of a ROI, along with a plot of relative strain values from FCC and HCP grains measured after irradiation (Fig. 3c).

In the grains which maintained their FCC structure after ion irradiation, compressive strains ranging from 1.1 to 1.8% normal strain (Max [ε_xx_, ε_yy_]) and 0.2–0.8% shear strain (ε_xy_) were measured. On the contrary, grains which transformed to HCP structure exhibited very low tensile strains varying from 0.1 to 0.2% normal strain (Max [ε_xx_, ε_yy_]) and 0.005–0.09% shear strain (ε_xy_)*.* This implies that the FCC to HCP phase transformation in NC gold is associated with a relaxation which occurs in metals to accommodate external strain when subjected to severe plastic deformation^4^. In this case, external strain is imparted with ion irradiation. First-principles calculations have found the cohesive energies for FCC gold (− 3.2064 eV/atom) and HCP gold (-3.2045 eV/atom) to be very comparable indicating that the formation of HCP gold can certainly be energetically favorable under the right conditions^34^. Measurements conducted by Geist et al.^35^ revealed that the relief of irradiation-induced stresses occurs preferentially in a direction perpendicular to the target surface, exhibiting an increase in interplanar spacings. Referencing the pre-irradiation orientation map (in the “Methods” section) and the post-irradiation phase map (Fig. 3b), notice that all the grains which are oriented parallel to the [111] zone axis before ion bombardment exhibited the HCP after irradiation; it is apparent that HCP phase is liable to form on the {111} planes which are least inclined to the sample surface, a result which may help explain why the formation of HCP is favored on planes with a particular orientation with respect to the sample surface.

The difference between FCC and HCP crystal structures is the stacking sequence amid atomic planes; HCP atomic layers cycle between two equivalent shifted positions whereas FCC layers cycle between three positions. HCP contains only two stacking positions of planes, A and B, with an alternating “ABABAB”… arrangement with the atoms of every other plane positioned exactly the same. However, the FCC structure contains three different stacking positions of planes with an “ABCABCABC”… arrangement. Dissimilar to HCP, the atoms in stacking sequence A and C do not align. The FCC cubic lattice structure allows slippage to occur more easily than non-cubic lattices, hence HCP metals (with 2 independent slip systems) are less ductile than FCC metals (with 12 slip systems)^36^.

It is difficult to transform “ABCABC”… stacking to “ABABAB”… stacking as moving one complete stacking sequence of FCC would require a lot of energy. One possible route for this sort of transformation can be accomplished by motion of **a/6** $\left\langle {112} \right\rangle$ dislocations on alternating slip planes^37^. Therefore, it is asserted that the activation barrier for the phase transformation is overcome via strain energy provided by the ion irradiation-induced defects thus the compressive strains greater than 1.8% in FCC gold leads to the phase transformation to HCP gold.

**Figure 4:**
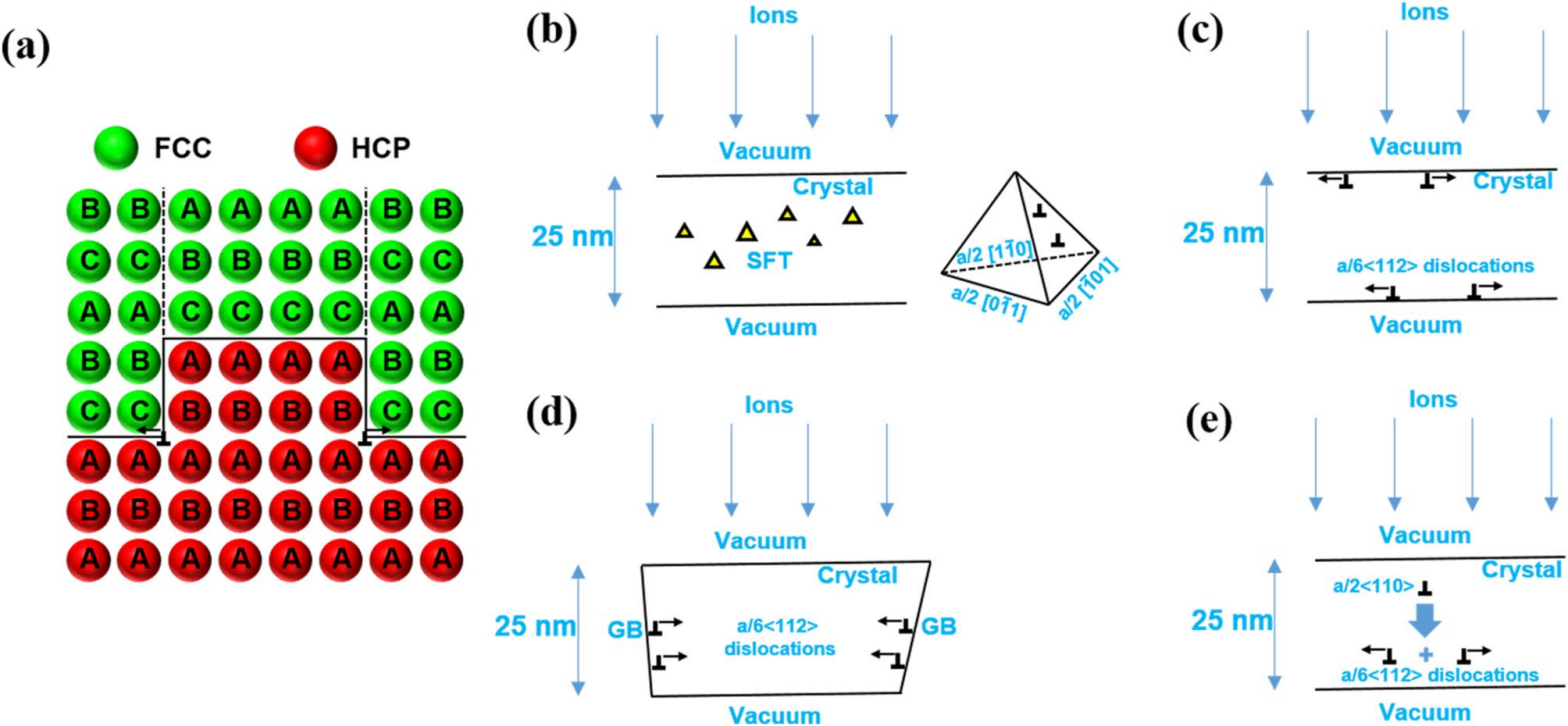
Proposed mechanisms of FCC to HCP transformation. Schematic showing a change in the packing of the atomic layers from “ABC” (FCC) to “AB” (HCP) and the various possible nucleation sites for the transformation. (**a**) Propagation of **a/6** $\left\langle {112} \right\rangle$ Shockley partials transforming two layers of FCC to HCP. (**b**) Formation of SFT with ion irradiation and nucleation of **a/6** $\left\langle {112} \right\rangle$ Shockley partials on the SFT faces. Nucleation of **a/2** $\left\langle {110} \right\rangle$ dislocations at the (**c**) crystal-vacuum interface and (**d**) GB. This is followed by dissociation into **a/6** $\left\langle {112} \right\rangle$ Shockley partials and their propagation through the crystal with ion irradiation at 200 °C. (**e**) Nucleation of **a/2** $\left\langle {110} \right\rangle$ dislocations in the crystal matrix followed by dissociation into **a/6** $\left\langle {112} \right\rangle$ Shockley partials and their propagation through the crystal with ion irradiation at 200 °C.

Furthermore, the atomistic mechanism that could contribute towards the phase transformation of FCC (“ABC” stacking) to HCP (“AB” stacking) in NC gold is probed and possible nucleation sites for the transformation are enumerated in Fig. 4. The proposed mechanism is depicted via schematic in Fig. 4a, which is guided by the experimental results and atomic model of Howe et al.^38^ for the growth of HCP precipitates inside FCC matrix. In FCC metals, dislocations of the **a/2** $\left\langle {110} \right\rangle$ character form relatively easily with deformation on {111} planes. Since gold has a relatively low SFE of 30 mJ/m^2^, it is expected that upon ion irradiation a large number of **a/2** $\left\langle {110} \right\rangle$ dislocations nucleate inside the FCC grains which further dissociate into two **a/6** $\left\langle {112} \right\rangle$ Shockley partials. These Shockley partials propagate throughout the grain with continuous ion irradiation at 200 °C. This passage of Shockley partials transforms the two layers of FCC into HCP. It is expected that the inherently present **a/2** $\left\langle {110} \right\rangle$ dislocations also break into Shockley partials and become more active with ion irradiation at 200 °C. This kind of FCC to HCP transformation via propagation of **a/6** $\left\langle {112} \right\rangle$ Shockley partials was observed via high-resolution TEM in Al-Ag alloys where the dissemination of Shockley partials caused the growth of HCP precipitates inside the FCC matrix^38^. These Shockley partials are glissile at room temperature in both FCC and HCP phases therefore the rapid migration of these dislocations inside the crystal at the ion irradiation temperature of 200 °C is expected.

Possible nucleation sites for the Shockley partials are further reviewed here: since defects are readily induced in metals, point, line, and defect clusters, as well as interfaces are expected to serve as favorable nucleation sites for the propagation of the phase transformation. We propose that: (Fig. 4b–e) (b) surface of SFT, (c) crystal-vacuum interface, (d) GB, and (e) crystal matrix are the most likely nucleation sites to initiate Shockley partials motion and hence are the most probable nucleation sites for the phase transformation. {111} SFT faces favor the dissociation of **a/2** $\left\langle {110} \right\rangle$ dislocations into **a/6** $\left\langle {112} \right\rangle$ Shockley partials, therefore, SFT faces can easily serve as the heterogeneous nucleation site for phase transformation (Fig. 4b)^39^; discusses potential mechanisms of SFT as a source of stacking faults. GBs emit **a/2** $\left\langle {110} \right\rangle$ dislocations frequently in FCC crystals which can further dissociate into **a/6** $\left\langle {112} \right\rangle$ Shockley partials and pass through the crystal for transformation (Fig. 4d) forming HCP at GBs as described by Medlin et al.^40,41^. Similarly, **a/2** $\left\langle {110} \right\rangle$ dislocations can nucleate at the crystal-vacuum interface (Fig. 4c) and inside the crystal matrix (Fig. 4e) followed by dissociation into **a/6** $\left\langle {112} \right\rangle$ Shockley partials and their propagation.

A model for the FCC to HCP transformation was proposed by Mahajan, Green, and Brasen^37^: the dislocation reaction **a/2** $\left\langle {1\overline{1}0} \right\rangle$ + **a/2**$\left\langle {10\overline{1} } \right\rangle$  → 3 × **a/6**$\left\langle {2\overline{1}\overline{1}} \right\rangle$ initiates the process, then when these nuclei located at different levels grow into each other a macroscopic HCP region is formed. This model is evidenced by a comprehensive TEM study on deformation induced formation of HCP in 18/8-type austenitic stainless steel where observations were carried out on the formation and processes of stacking faults under stress^42^. As reviewed by Fujita and Ueda, it is conventionally understood that lattice imperfections, namely the overlapping of stacking faults, are involved in the FCC to HCP transformation. Although extended bands of stacking faults were not readily identifiable due to intense contrast in our heavily damaged irradiated Au specimen, the presence of ample SFTs evidently implies the presence of stacking faults in concentration. These SFTs, numerous and in close proximity, provide a fault arrangement capable of fostering dislocation interactions which ultimately nucleate HCP. Fujita and Ueda concluded that HCP crystals can be formed by the following methods: (a) Wide stacking faults are formed by either (1) interaction among dislocations or (2) cross-slip of stair-rod type. (b) Neighboring slip planes of the wide stacking faults are easily activated, on which many of active dislocations are widely extended and overlap each other. (c) Further stacking faults tend to overlap each other on every two atomic layers; the overlapping of stacking faults occurs irregularly at first, and then gradually changes to the regular sequence. In the case of deformation, the overlapping of stacking faults occurs irregularly then gradually changes to a regular sequence. Namely, the stacking faults are in the FCC matrix at first and then further stacking faults are easily induced on {111} planes near the original fault planes due to the minimization of the bulk free energy and the total energy of stacking faults. Consequently, stacking faults are usually arranged on every second slip plane so that the HCP phase structure is formed. Observations of extended defects adjacent to crystal interfaces also indicated that faults could nucleate from imperfections present in the boundaries.

Localized sample thickness gradients are another consideration for the phase transformation inconsistencies. In a study identifying HCP nanoparticles in rhodium researchers employed first-principle calculations to estimate the surface energies of planes in FCC and HCP lattices^14^. Several low-index FCC and HCP surfaces were studied; for HCP rhodium, there are two low energy surfaces, (0001) and (101$\overline{0}$), while (111) is the only one for FCC. Their surface energies have the order (0001) HCP < (111) FCC < (101$\overline{0}$) HCP, therefore, from the surface energy point of view, small size can stabilize HCP Rh, which has a lower energy surface relative to FCC. Surface energies show different degrees of size dependence hence foil thickness is an imperative characteristic affecting specimen properties the nanoscale. Our low-loss EELS data showed an 18% variation in sample thickness which correlates to ~  ± 5 nm compared to the average foil thickness, a difference sufficient to affect structural and chemical properties. Further work is necessary to deconvolute the direct effects of film thickness, grain size, and displacement damage on the overall observed phase transformation.

## Conclusion

This study highlights the possible mechanisms governing the irradiation-induced FCC to HCP phase transformation in NC gold thin-films. Heavy ion bombardment imparts cascade damage resulting in microstructural evolution, which was documented using of a combination of in situ irradiation, atomic-resolution transmission electron microscopy, and precession electron diffraction techniques. It was found that a significant portion (~ 84% of the ROI) of the specimen underwent a phase transformation exhibiting a preferential {111}_FCC_||{0001}_HCP_ orientation relationship. This phenomenon is proposed as a unique result stemming from nanograined processing, irradiation-induced deformation, as well as thin-film and surface effects. The proposed mechanism is offered as a unique route for the stabilization of HCP gold nanostructures. Furthermore, the results give evidence that upon exceeding a particular strain threshold introduced by radiation-induced defects, stress relaxation occurs via phase transformation. Further work is underway to full elucidate the factors governing this transformation and the potential impact.

## Methods

### Specimen preparation

**Figure 5:**
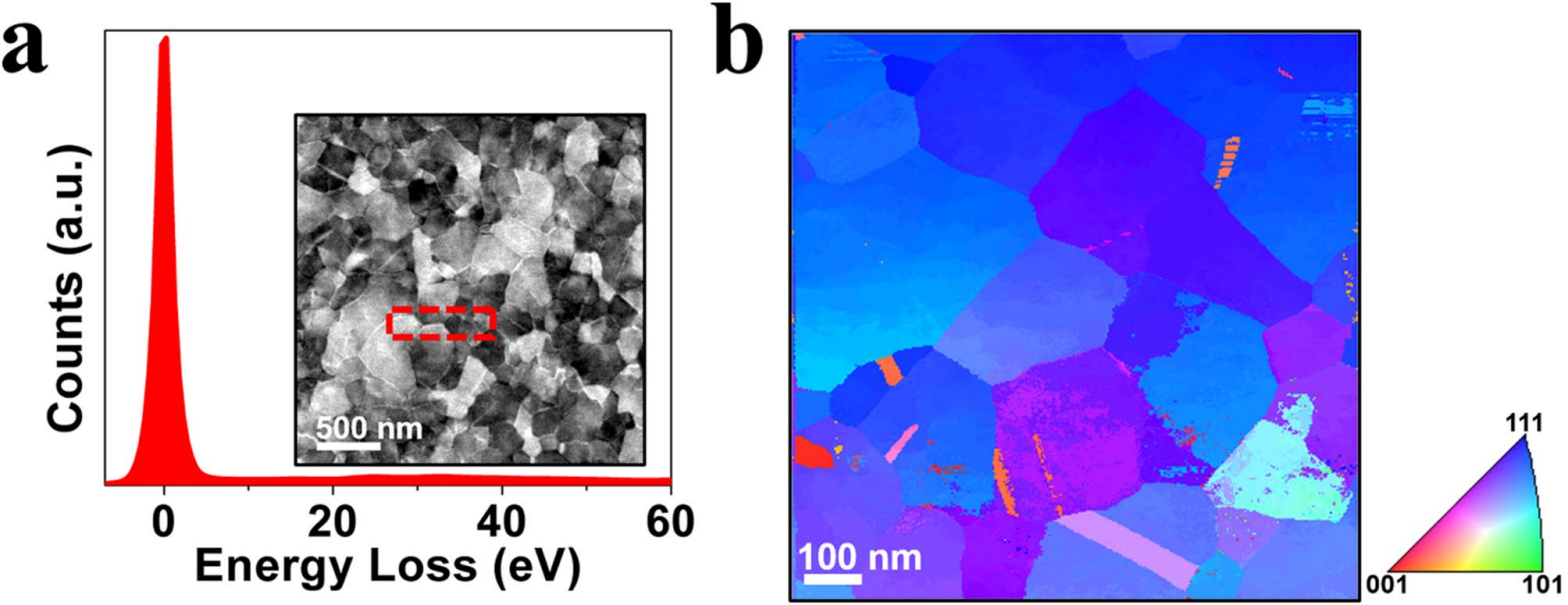
Gold thin-film specimen characterization. (**a**) Representative low-loss EEL spectrum used to determine crystal thickness; inset shows a representative HAADF-STEM image of the gold film, the dashed red box denotes the region for low-loss EEL spectrum acquisition. (**b**) Precession electron diffraction map from the NC gold film before ion irradiation with inset inverse pole figure key.

A thin-film of gold was sputter deposited on NaCl substrates, then floated onto a Mo TEM mesh grid in a 50/50-water/ethanol solution. The sample was then annealed at 625 °C for 30 min in a JEOL 2100F TEM using a Gatan 628 heating holder. In situ annealing was used to actively monitor grain growth to maintain a nanocrystalline microstructure. The thickness of the film was estimated to be 25 nm from a t/λ map/zero-loss peak acquired from energy filtered TEM (EF-TEM) of the area of interest shown (Fig. 5a); the film thickness was found by comparing the intensity of the zero-loss peak of the low-loss region out to 60 eV in the EEL spectra^43^ and calculating the inelastic mean-free path of a 200 keV electron in gold as 58 nm^44^.

### Characterization and irradiation

Pre- and post-irradiation TEM characterization including energy filtered TEM and precession electron diffraction (PED) were performed using a JEOL 2100F TEM operated at 200 kV. NanoMegas ASTAR precession electron diffraction with TopSpin acquisition system was used for crystal orientation and relative strain mapping. Precession angles of 0.6° and 1° were used for the orientation and strain mapping, respectively. Via orientation mapping, it was determined, as expected, that the annealed film had a $\left\langle {111} \right\rangle$ out-of-plane texture (Fig. 5b) as most of the grains were oriented close to the $\left\langle {111} \right\rangle$ zone axis in the direction normal to the electron beam. The region of interest (ROI) selected for stain analysis had an average grain size of ~ 250 nm with grains ranging from 73 to 451 nm.

Atomic-resolution TEM images were also captured before and after irradiation with an image C_s_-corrected FEI Titan G2 (S)TEM operated at 300 kV; Fourier filtering was used to enhance signal-to-noise ratio in the atomic-resolution images. In situ TEM irradiation experiments were performed at the I^3^TEM facility at Sandia National Laboratories. The annealed NC gold sample was irradiated with 2.8 meV Au^4+^ ions at 200 °C with a dose rate of 3.1 × 10^11^ ions/cm^2^/s to the final dose of 4.1 × 10^14^ ions/cm^2^ (approximately 10 dpa). During irradiation the sample stage was tilted 30° (relative to the electron beam) toward the ion beam resulting in a 60° incident angle between the ion beam and the sample surface. The projected range of the ions bombarding the sample was ~ 100 nm according to Stopping and Range of Ions in Matter (SRIM) 2013^45^, implying that the vast majority of Au^4+^ ions pass through the sample without implantation. A value of 40 eV was used for the displacement damage energy of Au^46^ and the peak damage depth was calculated to be within the sample thickness at 20 nm beyond the surface of beam entry (Fig. S2 in Supplementary Section). A sputtering yield ((number of sputtered atoms)/(number of incident atoms)) of 0 was calculated per SRIM. Based on this sputtering yield assessment from SRIM, we have concluded that sputtering is not an extensive factor contributing to the sample’s final thickness and crystal structure.

## Supplementary information

Supplementary information.

## Data Availability

The datasets generated during and/or analyzed during the current study are available from the corresponding author on reasonable request.
