# Supplementary material for: Ion irradiation induced phase transformation in gold nanocrystalline films
Source: Sci Rep. 2020 Oct 20;10:17864. doi: 10.1038/s41598-020-74779-2 (PMC7576776; doi:10.1038/s41598-020-74779-2)
Supplement: Supplementary file 1 — Supplementary information. [file 41598_2020_74779_MOESM1_ESM.docx]

Supporting Information

**Ion Irradiation Induced Phase Transformation in Gold Nanocrystalline Films**

Pranav K. Suri^1,†^, James E. Nathaniel, II^1,2^, Nan Li^3^, Jon K. Baldwin^3^, Yongqiang Wang^4^, Khalid Hattar^5^, Mitra L. Taheri^1,2^*

*^1^Department of Materials Science and Engineering, Drexel University, Philadelphia, PA, USA*

*^2^Department of Materials Science and Engineering, Johns Hopkins University, Baltimore, MD, USA*

*^3^Ceter for Integrated Nanotechnologies, MPA Division, Los Alamos National Laboratory, Los Alamos, NM, USA*

*^4^MST-8, Los Alamos National Laboratory, Los Alamos, NM, USA*

*^5^Sandia National Laboratories, Albuquerque, NM, USA*

†Present Address: Micron Technology, Inc., Boise, ID, USA


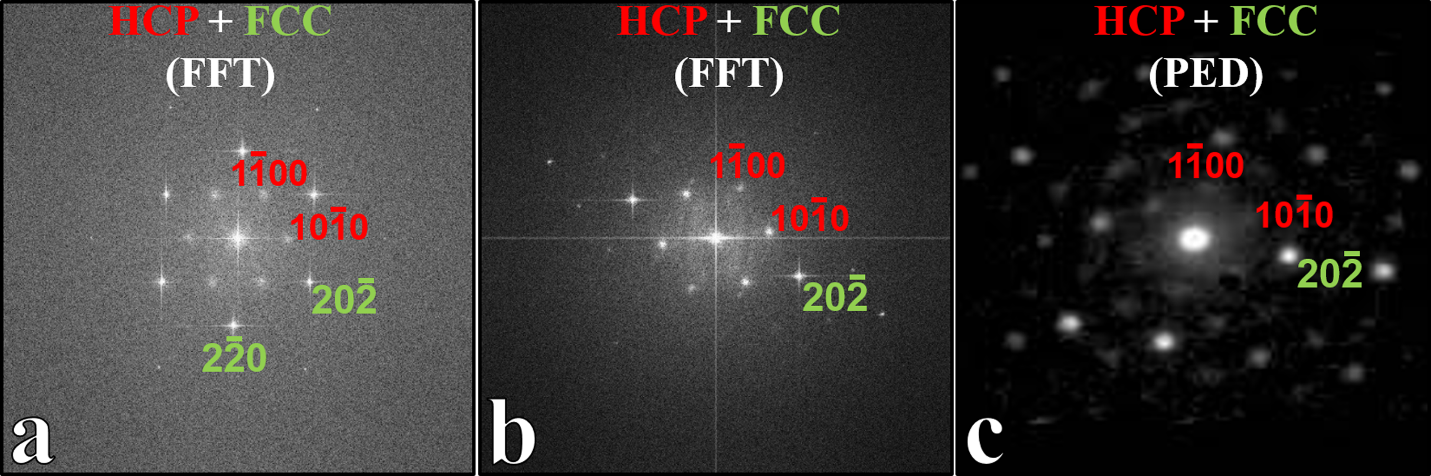
**Figure S1. Fast-Fourier Transform (FFT) images and Precession Electron Diffraction (PED) pattern showing both HCP and FCC phases after ion irradiation** **in selected regions of the sample** (a,b) FFT images and (b) PED pattern showing HCP reflections in red and FCC reflections in green. Note that these images and patterns are from the regions of sample where there is incomplete phase transformation.


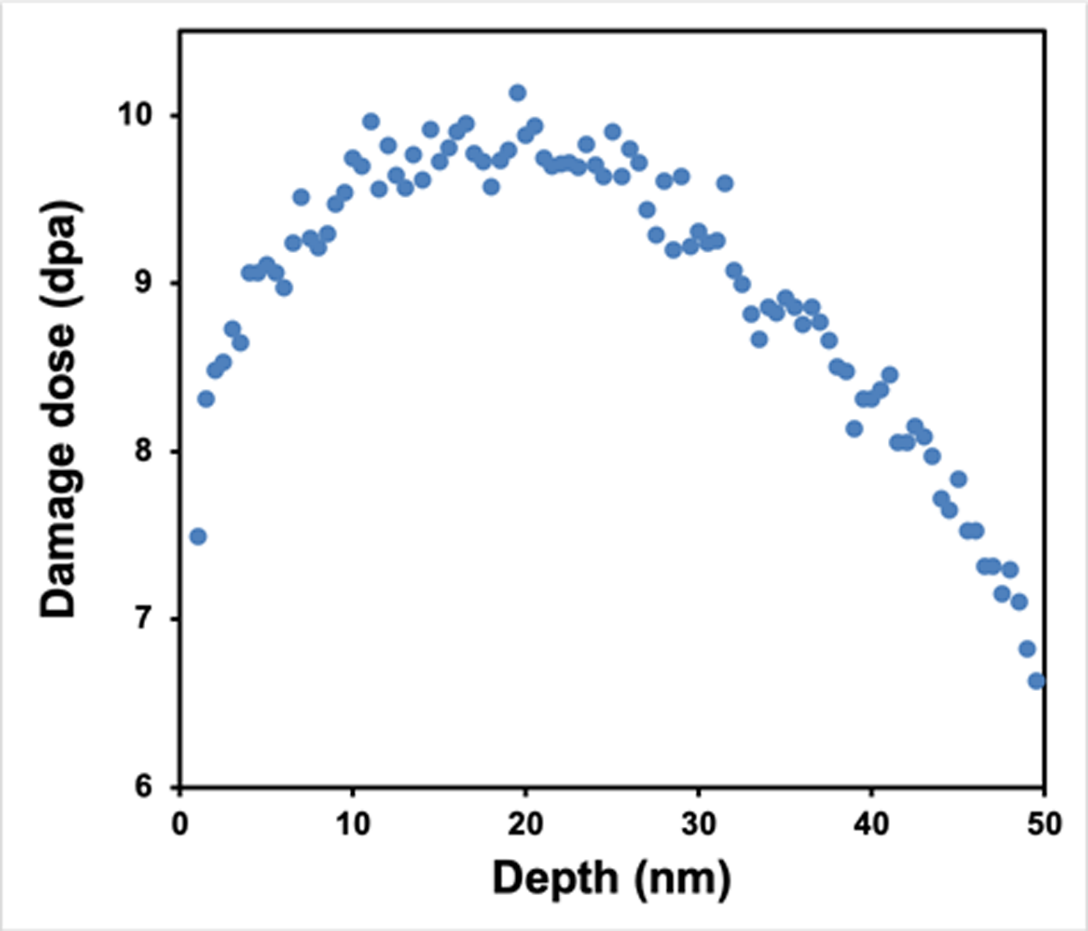


**Figure S2. Damage dose and specimen depth correlation for the current experimental setup.**
